# Extracellular superoxide dismutase is necessary to maintain renal blood flow during sepsis development

**DOI:** 10.1186/s40635-017-0130-9

**Published:** 2017-03-16

**Authors:** Larissa Constantino, Letícia Selinger Galant, Francieli Vuolo, Karla Lorena Guarido, Luiza Wilges Kist, Giovanna Medeiros Tavares de Oliveira, Matheus Augusto de Bittencourt Pasquali, Cláudio Teodoro de Souza, José Eduardo da Silva-Santos, Maurício Reis Bogo, José Cláudio Fonseca Moreira, Cristiane Ritter, Felipe Dal-Pizzol

**Affiliations:** 1 Laboratório de Fisiopatologia Experimental, Universidade do Extremo Sul Catarinense, Avenida Universitária, 1105, 88806-000 Criciúma, SC Brazil; 2 Departamento de Farmacologia, Laboratório de Biologia Cardiovascular, Universidade Federal de Santa Catarina, Campus Trindade, CEP 88040-900 Florianópolis, SC Brazil; 3 Laboratório de Biologia Genômica e Molecular, Faculdade de Biociências, Pontifícia Universidade Católica do Rio Grande do Sul, Avenida Ipiranga, 6681, 90619-900 Porto Alegre, RS Brazil; 4 Departamento de Bioquímica, Centro de Estudos em Estresse Oxidativo (Lab. 32), ICBS, Universidade Federal do Rio Grande do Sul, Rua Ramiro Barcelos, 2600-Anexo, CEP 90035-003 Porto Alegre, RS Brazil; 5 Laboratório de Fisiologia e Bioquímica do Exercício, Universidade do Extremo Sul Catarinense, Avenida Universitária, 1105, 88806-000 Criciúma, SC Brazil

**Keywords:** Sepsis, Acute renal failure, Superoxide, Nitric oxide

## Abstract

**Background:**

Extracellular superoxide dismutase (ECSOD) protects nitric oxide (NO) bioavailability by decreasing superoxide levels and preventing peroxynitrite generation, which is important in maintaining renal blood flow and in preventing acute kidney injury. However, the profile of ECSOD expression after sepsis is not fully understood. Therefore, we intended to evaluate the content and gene expression of superoxide dismutase (SOD) isoforms in the renal artery and their relation to renal blood flow.

**Methods:**

Sepsis was induced in Wistar rats by caecal ligation and perforation. Several times after sepsis induction, renal blood flow (12, 24 and 48 h); the renal arterial content of SOD isoforms, nitrotyrosine, endothelial and inducible nitric oxide synthase (e-NOS and i-NOS), and phosphorylated vasodilator-stimulated phosphoprotein (pVASP); and SOD activity (3, 6 and 12 h) were measured. The influence of a SOD inhibitor was also evaluated.

**Results:**

An increase in ECSOD content was associated with decreased 3-nitrotyrosine levels. These events were associated with an increase in pVASP content and maintenance of renal blood flow. Moreover, previous treatment with a SOD inhibitor increased nitrotyrosine content and reduced renal blood flow.

**Conclusions:**

ECSOD appears to have a major role in decreasing peroxynitrite formation in the renal artery during the early stages of sepsis development, and its application can be important in renal blood flow control and maintenance during septic insult.

## Background

Sepsis is a complex syndrome characterized by an imbalance between pro-inflammatory and anti-inflammatory responses to a pathogen [1, 2]. During its development, a large amount of reactive oxygen species (ROS) and nitric oxide (NO) are produced [3, 4]. In this context, superoxide radicals can react with NO to produce peroxynitrite [5, 6]. Peroxynitrite is a powerful oxidizing agent that is more cytotoxic than NO and is implicated in endothelial dysfunction [7].

The control of peroxynitrite levels and the bioavailability of NO depend, at least in part, on SOD activity [5, 6]. Superoxide dismutases (SODs) are metalloenzymes that catalyse the dismutation of the superoxide radical to hydrogen peroxide and oxygen. Mammals have three distinct isoforms of SOD; copper/zinc-SOD (Cu/ZnSOD, SOD1), manganese-SOD (MnSOD, SOD2) and extracellular (ECSOD, SOD3). ECSOD is predominantly localized in the extracellular matrix and the extracellular fluids depending on the presence of a carboxy-terminal heparin-binding domain [5, 6]. ECSOD is secreted by various cells and binds to glycosaminoglycans in the vascular extracellular matrix [8–10], maintaining NO bioavailability [11, 12]. Since the production of peroxynitrite is associated with decreased renal blood flow and acute kidney injury [13, 14], ECSOD could play a major protective role in this context, but little is known about its modulation during sepsis.

Recently, we have demonstrated the role of ECSOD in lung injury after sepsis development [15]. In a second set of experiments, we hypothesized that sepsis induces a decrease in ECSOD levels in the renal artery and that this effect is associated with nitrosative stress that decreases global renal blood flow and promotes acute kidney injury.

## Methods

### Animals

Male Wistar rats (350–400 g) were obtained from our own breeding colony. They were caged in groups of five with free access to food and water and were maintained on a 12-h light–dark cycle (6:00–18:00 h) in a temperature-controlled colony room (22 ± 1 °C). These conditions were maintained constantly throughout the experiments. The research protocol was approved by the Ethical Committee for Animal Experimentation of Universidade do Extremo Sul Catarinense under protocol number 21/2011, and the procedures are in accordance with the National Institutes of Health guidelines for animal care.

### Caecal ligation and perforation (CLP) surgery

The animals were subjected to CLP as previously described [15]. Briefly, rats were anaesthetized with ketamine and, under aseptic conditions, a 3-cm midline laparotomy was performed to allow for exposure of the caecum with the adjoining intestine. The caecum was tightly ligated with a 3.0-silk suture at its base, below the ileocaecal valve, and was perforated once with a 14-gauge needle. The caecum was then gently squeezed to extrude a small amount of faeces, returned to the peritoneal cavity, and the laparotomy was closed with 4.0-silk sutures. Animals were resuscitated with normal saline (50 mL/kg subcutaneous) immediately, 12 and 24 h after CLP, and were given antibiotics (ceftriaxone at 30 mg/kg and clindamycin 25 mg/kg) every 6 h and SC for the entire duration of the experiments. In a set of experiments, a non-specific SOD inhibitor, diethyldithiocarbamic acid diethylammonium (DETC, i.v. 7.5 mg/kg) [16, 17], was administered immediately after sepsis induction. All animals were returned to their cages with free access to food and water. In the sham-operated group, the rats were submitted to all surgical procedures, but the caecum was neither ligated nor perforated. All animals were observed and developed signs of infection (piloerection, lethargy, tachypnea or weight loss). The number of animals that survived is consistent with our previous reports.

### Direct measurement of renal blood flow

In this set of experiments, the animals were anaesthetized with ketamine/xylazine (90/15 mg/kg, i.m.) and a transverse abdominal incision was performed to assess the posterior right subhepatic space, allowing for the visualization of the right kidney. A laser probe (model VP1) connected to a laser Doppler blood flow monitor (moorVMS-LDF2, Moor Instruments, England) was carefully placed directly onto the kidney, allowing for the measurement of renal blood flow (in arbitrary units). The probe was kept in this position, and the surgical incision was covered with gauze sponges soaked in sterile phosphate-buffered saline to protect the kidney from drying out. The laser probe remained tightly fixed on the kidney surface, and very stable and constant trace recordings of renal blood flow were obtained. During the experiments, the animals were maintained on a warming pad and allowed to breathe spontaneously. An interval of 20 min was observed before the measurement of basal values and renal blood flow was measured for an additional 15 min [18].

### Renal artery extraction and sample preparation

The animals were anaesthetized with ketamine and killed by decapitation at different times after surgery. The renal arteries were dissected out under ×4 magnification on ice and stored at −80 °C for posterior analyses.

### Total SOD activity

SOD activity was measured by the inhibition of adrenaline auto-oxidation followed spectrophotometrically as previously described [19].

### Immunoblotting

Samples were lysed in Laemmli buffer (62.5 mM Tris-HCl, pH 6.8, 1% (*w/v*) SDS, 10% (*v/v*) glycerol), and equal amounts of protein were fractionated by SDS-polyacrylamide gel electrophoresis (SDS-PAGE) and electroblotted onto nitrocellulose membranes. Protein loading and electroblotting efficiency were verified through Ponceau S staining, and the membrane was blocked in Tween–Tris-buffered saline (TTBS: 100 mM Tris-HCl, pH 7.5, containing 0.9% NaCl and 0.1% Tween-20) containing 5% albumin. Membranes were incubated overnight at 4 °C with rabbit polyclonal antibodies against SOD1 (1:400), SOD2 (dilution range, 1:400), SOD3 (dilution range, 1:750), iNOS (1:750), eNOS (1:750), pVASP (1:600) or *β*-actin (1:2000) and then washed with TTBS. Anti-rabbit immunoglobulin G (IgG) peroxidase-linked secondary antibody was incubated with the membranes for 1 additional hour (1:10000 dilution range). The membranes were washed again, and the immunoreactivity was detected by enhanced chemiluminescence using an ECL Plus kit. Densitometric analysis of the films was performed with ImageQuant software. Blots were developed to be linear in the range used for densitometry. All results were expressed as relative ratios between SOD1, SOD2, SOD3, iNOS, eNOS and pVASP immunocontent and the *β*-actin internal control immunocontent.

### Analysis of gene expression by semi-quantitative RT-PCR

All transcriptional analyses were performed in samples that were different between groups in the immunocontent in the blotting analysis with the goal of evaluating the contribution of each gene to the immunocontent of each enzyme. Total RNA was isolated from rat renal arteries using TRIzol^®^ reagent (Invitrogen, Carlsbad, CA, USA) in accordance with manufacturer instructions. The purity of the RNA was spectrophotometrically quantified by calculating the ratio between absorbance values at 260 and 280 nm, and its integrity was confirmed by electrophoresis through a 1.0% agarose gel. Afterwards, cDNA species were synthesized using ImProm-II™ Reverse Transcription System (Promega^®^) following the supplier's instructions. The cDNA products (1 μL) were used as a template for each PCR amplification. PCR parameters were first optimized, and then reactions were performed allowing for product detection within the linear phase of mRNA transcript amplification for each primer pair. PCR for the *β-actin* gene was performed in a total volume of 20 μL using 0.1 μM of each primer, 0.2 μM dNTP, 1.6 mM MgCl_2_ and 0.2 U Taq platinum DNA polymerase (Invitrogen). In the PCR for *SOD1, SOD2 and SOD3*, the reaction was performed in a total volume of 25 μL using 0.2 μM of each primer, 0.2 μM dNTP, 1.6 mM MgCl_2_ and 0.25 U Taq platinum DNA polymerase (Invitrogen). Conditions for *sod 1*, *sod 2* and *sod 3* PCR were as follows: an initial 1-min denaturation step at 94 °C; 1 min at 94 °C, a 1-min annealing step at 60 °C, and a 1-min extension step at 72 °C for 30 cycles; and a final 10 min extension at 72 °C. Conditions for *β-actin* PCR were as follows: an initial 1-min denaturation step at 94 °C; 1 min at 94 °C, a 1-min annealing step at 54 °C, and a 1-min extension step at 72 °C for 35 cycles; and a final 10-min extension at 72 °C. For each PCR set, a negative control was included. PCR products were analysed on a 1% agarose gel containing GelRed^®^ and visualized with ultraviolet light. The Low DNA Mass Ladder (Invitrogen) was used as a molecular marker, and samples were normalized by employing *β-actin* as a constitutive gene. The band intensities were measured by optical densitometry analysis, and the *enzyme*/*β-actin* mRNA ratios were established for each treatment using the freeware Image J 1.37. Each experiment was repeated at least four times using RNA isolated from independent extractions.

### Enzyme-linked immunosorbent assay (ELISA) for 3-nitrotyrosine contents

An indirect ELISA assay was performed according to the manufacturer’s instructions. Briefly, microtiter plates were coated for 24 h with the samples diluted 1:2 in PBS with 5% albumin. Plates were then washed four times with wash buffer (PBS with 0.05% Tween-20), and the antibody was added to each plate for 2 h at room temperature. After washing, a second incubation with anti-rabbit antibody peroxidase conjugated (diluted 1:1000) for 1 h at room temperature was carried out. After the addition of substrates (hydrogen peroxide and tetramethylbenzidine 1:1, *v*/*v*), the samples were read at 450 nm in a plate spectrophotometer.

### Statistical analysis

Data are expressed as the mean ± S.E.M and compared by the independent *t* test or ANOVA followed by the Tukey post hoc test depending on the sample characteristics. Statistical significance was set at *p <* 0.05.

## Results

**Fig. 1:**
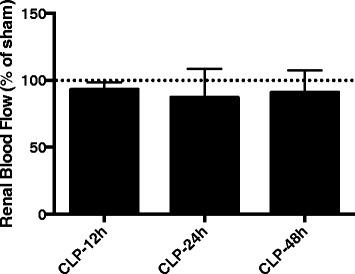
Renal blood flow after sepsis induction. Sepsis was induced, and renal blood flow was measured at 12, 24 and 48 h. The *dotted line* represents sham renal blood flow. The values are presented as the mean ± S.E.M., *n* = 6 in each group

**Fig. 2:**
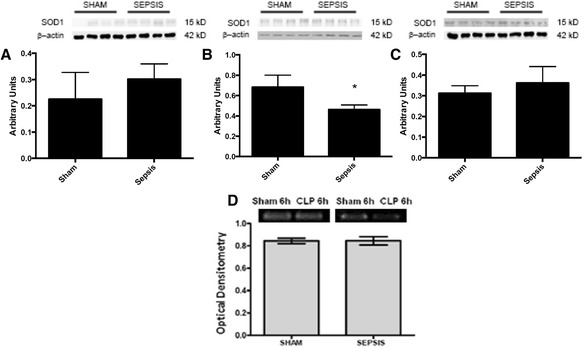
Superoxide dismutase 1 (SOD1) immunocontent and gene expression after sepsis induction by CLP. Three times, 3 h (**a**), 6 h (**b**) and 12 h (**c**), after sepsis, the renal arteries were isolated, and the content of SOD1 was quantified by immunoblot. Representative western blots show samples from shams in the first bands and samples from sepsis in the last bands, represented in equal number. The densitometric analysis quantification is depicted in *bar graphs*. Gene expression of SOD1 was measured by RT-PCR, with b-actin as control, 6 h (**d**) after the induction of sepsis. The results are expressed as optical densitometry for the mean ± S.E.M. (*n* = 6 animals each group). *p < 0.05 compared to the sham group

**Fig. 3:**
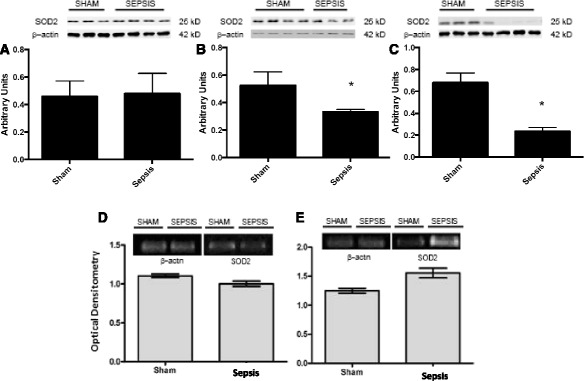
Superoxide dismutase 2 (SOD2) immunocontent and gene expression after sepsis induction by CLP. Three times, 3 h (**a**), 6 h (**b**) and 12 h (**c**), after sepsis, the renal arteries were isolated, and the content of SOD2 was quantified by immunoblot. Representative western blots show samples from shams in the first bands and samples from sepsis in the last bands, represented in equal number. The densitometric analysis quantification is depicted in *bar graphs*. Gene expression of SOD2 was measured by RT-PCR, with b-actin as control, 6 h (**d**) and 12 h (**e**) after the induction of sepsis in sham and sepsis groups. The results are expressed as optical densitometry for the mean ± S.E.M. (*n* = 6 animals in each group). *p < 0.05 compared to the sham group

**Fig. 4:**
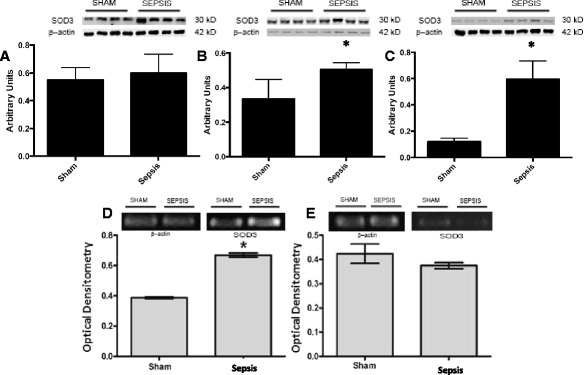
Superoxide dismutase 3 (SOD3) immunocontent and gene expression after sepsis induction by CLP. Three times, 3 h (**a**), 6 h (**b**) and 12 h (**c**), after sepsis, the renal arteries were isolated, and the content of SOD3 was quantified by immunoblot. Representative western blots show samples from shams in the first bands and samples from sepsis in the last bands, represented in equal number. The densitometric analysis quantification is depicted in *bar graphs*. Gene expression of SOD3 was measured by RT-PCR, with b-actin as control, 6 h (**d**) and 12 h (**e**) after the induction of sepsis in sham and sepsis groups. The results are expressed as optical densitometry for the mean ± S.E.M. (*n* = 6 animals in each group). *p < 0.05 compared to the sham group

In this model, renal blood flow was maintained at early stages after sepsis induction (Fig. 1). Compared to sham animals, renal blood flow remained unchanged at least until 48 h after CLP (Fig. 1). Since SOD is a factor in renal blood flow control, we further investigated the protein content and expression of SOD in the renal arteries. Cu/ZnSOD protein content decreased at 6 h, but not at 3 or 12 h, after the induction of sepsis (Fig. 2a–c), which was not secondary to the downregulation of its gene expression (Fig. 2d). MnSOD content decreased from 6 to 12 h (Fig. 3a –c), but there was no downregulation of its gene expression (Fig. 3d, e). In contrast, ECSOD increased at 6 and 12 h after surgery (Fig. 4a –c), which could be partially secondary to an increase in its gene expression (Fig. 4d, e). The total SOD activity in the renal arteries was increased in septic animals when compared to sham animals at 6 and 12 h after sepsis (2.3 ± 0.9 vs 30.1 ± 9, *p* < 0.01; 2.6 ± 1.1 vs 34.1 ± 12, *p* < 0.01, respectively).

**Fig. 5:**
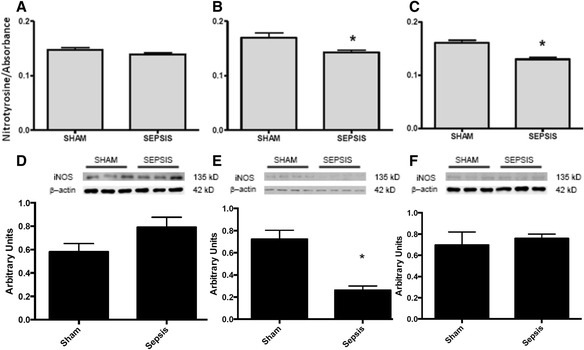
Levels of 3-nitrotyrosine by ELISA and iNOS expression by blotting after sepsis induction by CLP. At times 3 h (**a**), 6 h (**b**) and 12 h (**c**) after sepsis induction, the renal arteries were isolated, and the content of 3-nitrotyrosine was quantified by ELISA and expressed in terms of nitrotyrosine concentration/absorbance. The content of iNOS was measured by immunoblot 3 h (**d**), 6 h (**e**) and 12 h (**f**) after sepsis. Representative western blots showed samples from shams in the first bands and samples from sepsis in the last bands, represented in equal number. The densitometric analysis quantification is depicted in *bar graphs*. The results are expressed as the mean ± S.E.M. in arbitrary units. (*n* = 6 animals in each group). *p < 0.05 compared to the sham group

**Fig. 6:**
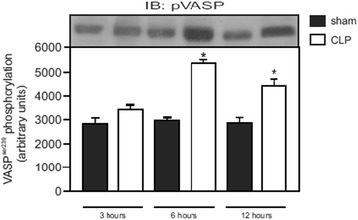
Levels of phosphorylated vasodilator-stimulated phosphoprotein (pVASP) expression after sepsis induction. Three times, 3 h, 6 h, and 12 h after sepsis, the renal arteries were isolated and the content of pVASP was quantified by immunoblot. Representative western blots are positioned above the correspondent bar group, in each sham and sepsis group, and densitometric analyses quantification is depicted in *bar graphs*. The results are expressed as the mean ± S.E.M. in VASP^Ser239^ phosphorylation (arbitrary units) (*n* = 6 animals in each group). *p < 0.05 compared to the sham group

We further measured the levels of nitrotyrosine, which decreased at 6 and 12 h after sepsis (Fig. 5a –c). However, the immunocontent of iNOS was decreased only 6 h after sepsis (Fig. 5d –f), and there were no differences in the content of eNOS between the groups and time (data not shown). To further evaluate the mechanisms involved in the preservation of renal blood flow, we analysed the content of pVASP, which increased at 6 and 12 h after sepsis induction (Fig. 6).

**Fig. 7:**
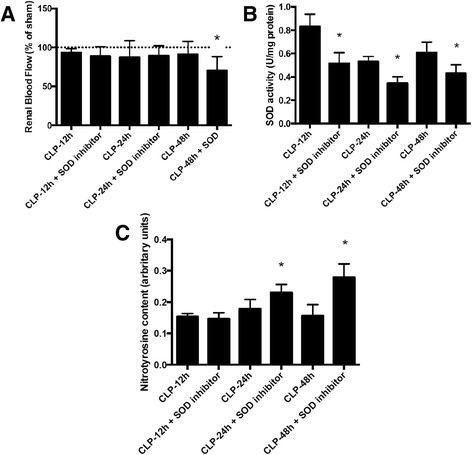
The effect of administration of a superoxide dismutase inhibitor on renal blood flow, superoxide dismutase activity and 3-nitrotyrosine content after sepsis induction. Immediately after sepsis induction, a SOD inhibitor, diethyldithiocarbamic acid diethylammonium (DETC, i.v. 7.5 mg/kg), was administered. Several times after sepsis induction (12, 24 and 48 h), renal blood flow was measured and (**a**) expressed as a percentage difference compared to the sham group; superoxide dismutase activity (**b**) was expressed in SOD (U/mg protein), and the 3-nitrotyrosine content (**c**) was expressed in nitrotyrosine content (arbitrary units). The *dotted line* represents sham renal blood flow. The values are presented as the mean ± S.E.M., *n* = 6 in each group. *p < 0.05, compared to CLP without SOD inhibitor at the same time point

To establish that the early increase in SOD content and activity was associated with the control of renal blood flow, a SOD inhibitor was injected early after sepsis. SOD inhibition induced a decrease in renal blood flow at 48 h after sepsis induction, but not at 12 and 24 h (Fig. 7a) and not in sham animals (data not shown). To determine whether this treatment truly interfered with SOD activity, we demonstrated that it induced a decrease in SOD activity (from 12 to 48 h after sepsis, Fig. 7b) but not in SOD protein content (data not shown). Since the working hypothesis proposes that SOD activity maintains NO bioavailability, we further measured the nitrotyrosine content that was increased by SOD inhibition from 24 to 48 h (Fig. 7c).

## Discussion

In a fluid-resuscitated model of sepsis, we have demonstrated that renal blood flow was maintained, at least in part, secondary to the upregulation of ECSOD and SOD activity in the renal arteries.

Acute kidney injury is a well-known, major complication in sepsis. In different models of sepsis, renal hypoperfusion occurs and is associated with the generation of peroxynitrite [20–22]. There are reports suggesting that peroxynitrite-induced PARP activation is involved in renal hypoperfusion and in impaired endothelium-dependent vasodilation [23]. In a model of endotoxaemia, ECSOD expression was decreased and was consequently associated with decreased renal blood flow [12, 24]. In general, in these models, the animals are not fully fluid resuscitated and antibiotic treatment is missing. Here, in a more clinically relevant model of sepsis, we demonstrated that renal blood flow could be maintained for at least 48 h, which is consistent with early goal-directed therapy to preserve microcirculation both in animal models and in humans [25–27]. In contrast, it was reported in a LPS model that, even when using fully balanced fluids, compromises in microvascular and renal functions occurred [28]. This unexpected result could be related to the regulation of NO production since peroxynitrite seems to be one of the most relevant mediators of sepsis-induced AKI [13, 20, 21].

SODs are the first defence system against superoxide anion radicals. Vascular smooth muscle cells secrete large amounts of ECSOD, and it is thought that these cells are the major source of the enzyme in the vascular wall [29]. Actually, ECSOD is widely expressed in the cardiovascular system and in the kidney [30], and it is critical to controlling 3-nitrotyrosine formation [31]. In addition, circulating microparticles from septic patients are able to increase ECSOD expression in the rat heart [32], which is consistent with our results. Therefore, despite the decreases in Mn and Cu/Zn SOD content after sepsis, there is an increase in total SOD activity that is probably associated with the observed increase in ECSOD. These results suggest that ECSOD is of major importance in maintaining kidney redox status early during sepsis. Actually, post-ischaemic renal vasoconstriction is associated with an impairment of endothelium-dependent NO-mediated vasodilation in renal vasculature [13, 33], but ECSOD overexpression prevents endothelial dysfunction and preserves blood flow [34]. It has also been demonstrated that a SOD mimetic decreased superoxide formation in the vascular compartment [35], which is consistent with our results. The maintenance of renal blood flow is probably mediated by SOD quenching of superoxide, causing a consequent decrease in peroxynitrite and improvement in vasorelaxation [36].

Therefore, if ECSOD is critical in regulating the redox state in the renal artery after sepsis, it can be expected that it could decrease 3-nitrotyrosine formation, which was, in fact, demonstrated in our model. Therefore, we propose that there is a decrease in the availability of superoxide early after sepsis due to an increase in ECSOD content, which in turn preserves NO levels to maintain renal blood flow. This is further supported by the fact that microparticles from septic patients did not change superoxide anion and nitric oxide production in the rat kidney [32]. It is suggested that, even without any detectable increase in NOS content, an increase in ECSOD would be sufficient to maintain NO levels in conditions where superoxide production is increased, such as in sepsis. NO plays a role in cyclic GMP-mediated smooth muscle relaxation leading to the production of pVASP [37–39]. We demonstrated an increase in pVASP at 6 and 12 h after sepsis induction, which was temporally related to the increase in ECSOD and the decrease in 3-nitrotyrosine, suggesting that NO signalling is preserved at these early stages of sepsis. Similarly, Fisher 344 rats are able to increase ECSOD in response to exercise as opposed to Sprague-Dawley rats, which indicates protection against acute kidney injury induced by ischaemia-reperfusion [40]. To ascertain that ECSOD plays a critical role in the control of renal blood flow, we administered a SOD inhibitor to septic animals. Inhibition of SOD not only induced an increase in nitrotyrosine levels but also resulted in decreased blood flow in later periods (48 h) of sepsis. One intriguing fact is that the SOD inhibitor did not affect renal blood flow at 12 and 24 h after sepsis. Actually, since the SOD inhibitor effectively inhibited SOD activity after 12 h, it was expected that nitrotyrosine levels and renal blood flow would also change, which occurs at 48 h. This probably reflects a steady state between NO and superoxide that could be affected by several other factors not assessed in our study. For example, it is well known that arginase activity could decrease NO synthesis by competing with NOS for arginine [41], and it has been demonstrated that arginase activity is modulated during sepsis development [42]. In addition, the role of SOD in the development of acute kidney injury was recently demonstrated in humans [43]. In septic shock patients, higher erythrocyte SOD activity was independently associated with protection from acute kidney injury [43]. However, the protective effects of ECSOD are also observed in other different tissues during sepsis development [15, 44].

Some limitations must be highlighted. First, renal blood flow was measured at three time points after sepsis, but renal blood flow may vary over time. Therefore, every hour time point measures for quasi-continuous monitoring would be required. This procedure would require a large number of animals (for each time point) or prolonged anaesthesia and mechanical ventilation, which could result in the generation of a different bias that could interfere with the results. Although the timeline of such changes remains to be further investigated by means of approaches that allow for the continuous measurement of renal blood flow, our results provide insight into the pharmacological use of an ECSOD mimetic to prevent kidney damage associated with sepsis. Second, we did not directly measure NO levels; therefore, we cannot determine if ECSOD activity truly preserved NO levels in our model. This was only indirectly measured by nitrotyrosine levels, pVASP content and renal blood flow.

## Conclusions

In conclusion, ECSOD has a relevant role in decreasing ONOO^-^ formation in the renal artery during early sepsis development, which can be an important step in the preservation of renal blood flow. These results provide insight into the pharmacological use of an ECSOD mimetic to prevent kidney damage associated with sepsis.
